# Predicting oil contamination in water using machine learning on microbial compositions

**DOI:** 10.1371/journal.pone.0344571

**Published:** 2026-03-19

**Authors:** Tong Gao, Isaac Bigcraft, Stephen Techtmann, Issei Nakamura

**Affiliations:** 1 Department of Physics, Michigan Technological University, Houghton, Michigan, United States of America; 2 Department of Biological Sciences, Michigan Technological University, Houghton, Michigan, United States of America; Commonwealth Scientific and Industrial Research Organisation, AUSTRALIA

## Abstract

We present a compact and generative machine-learning framework that predicts oil contamination based on microbial community compositions from experimental samples. Our method combines dimensionality reduction with data augmentation and generative modeling to address high-dimensional, non-linear, and sparse microbial data. To reduce the 503-dimensional bacterial composition dataset, we compared three dimensionality reduction techniques: feature importance from random forest, principal component analysis (PCA), and t-distributed stochastic neighbor embedding (t-SNE). Feature importance outperformed PCA and t-SNE, improving predictive performance and identifying microbial species most strongly correlated with oil contamination. To mitigate data scarcity, we augmented the training data using an augmented data neural network (ADNN) with noise injection. Samples generated by a variational autoencoder (VAE) were used as controlled perturbations to probe model robustness during stress testing. Using the top 3–10 bacterial features, our model achieved an R² value of up to 0.99 in both training and stress testing for predicting oil contamination from microbial data. In a bottle-level hold-out evaluation (22 splits at an 80/20 bottle ratio), performance on held-out bottles was lower and variable (mean test R² = −0.150), indicating limited generalization within this cohort. These results should be interpreted as a feasibility demonstration requiring validation on larger independent datasets.

## Introduction

Oil contamination continues to be a persistent environmental challenge, with oil spills causing extensive damage to both marine and terrestrial ecosystems and leading to long-term environmental degradation [1,2]. Detecting and preventing oil contamination effectively is therefore critical for environmental protection and resource management. While detecting large scale spills is trivial, as it can be done visually, detecting small leaks and transient spill risks can be more challenging. Microorganisms play vital roles in ecosystem functioning and are sensitive to environmental disturbance. Microbial communities have been previously shown to respond to even trace amounts of oil, resulting in substantial change in microbial community composition. For instance, King et al. summarized how indigenous marine microbes rapidly bloom in response to oil and gas release [3], and Redmond et al. observed that Gulf microbial communities shifted toward hydrocarbon-degrading taxa following the Deepwater Horizon spill [4]. Other fields report that oil contamination can cause 10-fold increases in bacterial abundance and enrichment of known degraders [5]. In seawater microcosms contaminated with oil, blooms of *Pseudomonadales* and *Methylococcales* greatly exceeded even the normal dominant *Alcanivorax*, indicating rapid community restructuring [6]. Understanding these microbial responses to oil and predicting their behaviors under varying environmental conditions is essential for developing effective environmental monitoring, management, and remediation strategies, with potential applications for a range of other contaminants.

Nevertheless, microbial data pose unique challenges, including high dimensionality [7], sparsity, and non-linear relationships. Obtaining oil contamination data that reveal how various types of oil affect microbial communities is difficult, as accurately modeling the complex interactions among microbes, crude oil, and refined oil remains poorly understood. Furthermore, microbial community taxonomic compositions often vary substantially due to minor shifts in environmental parameters such as location, season, and temperature. Additionally, collecting water samples, incubating microbes in laboratory settings, and measuring microbe abundances can be time-consuming and technically challenging. Therefore, sophisticated analytical tools like machine learning models with strong predictive capabilities are essential for addressing these multidimensional issues and revealing complex patterns [8]. Unlike traditional ecological models, such as predator-prey models, which struggle to account for the complexity of numerous bacterial species and their unknown interactions, machine learning methods can effectively function as simplified models to capture microbial dynamics.

Over the past decade, machine learning has emerged as a powerful tool for analyzing complex microbial datasets and predicting environmental changes. Various studies have demonstrated the potential of machine learning models to identify microbial responses to environmental stimuli [9,10]. Predictive models trained on microbial community data have been used in human and environmental health, including predicting the presence of environmental contamination [11]. However, despite these advancements, the accuracy, generalizability and translation of these models remain limited. Many existing frameworks rely on complex classifiers that perform well on familiar samples but struggle to generalize to unseen environments. For example, one recent study analyzed 86 out of 1709 soil microbiome taxa to predict microbial community compositions, achieving the highest accuracy of 0.65 on test data [12]. This reflects the inherent challenges posed by high-dimensional, sparse, and site-specific data, which can hinder the development of robust and scalable machine-learning frameworks. These limitations highlight the need for innovative approaches, including augmented data models to enrich datasets, noise-injection methods to improve model robustness, and autoencoders to decode and interpret predicted information. Moreover, research leveraging microbial community data combined with environmental information to trace contamination sources, such as oil pollution, is still in its early stages.

Experimentally collected microbial datasets are often limited in size, making it challenging to identify trends or achieve accurate predictions. Traditional machine learning methods typically struggle with these small datasets, resulting in reduced predictive power and accuracy. Generative approaches have shown their effectiveness in small-sample biological domains. For example, generative adversarial network (GAN)-augmented data improved disease prediction performance in microbiome studies, increasing sensitivity and specificity [13], and boosting disease AUCs (Area Under the Curve) by ~30% in datasets with limited sample sizes [14]. However, GANs are computationally intensive, and their most efficient algorithms are designed largely for image-based data rather than high-dimensional microbe community compositions [15]. In addition, microbial datasets are inherently high-dimensional, sparse, and often contain far fewer samples than features, making it difficult for traditional machine learning algorithms to perform robustly [16,17]. Further exacerbating the problem, environmental communities have far more inter-sample diversity than most clinical microbiomes. However, when sample sizes are limited, synthetic data cannot substitute for independent real-world test data. Instead, generative models can be used to probe robustness, structure, and sensitivity of learned representations under controlled perturbations.

To address these issues, we construct a neural network framework that integrates a generative model, variational autoencoder (VAE), and predictive neural network. It has been demonstrated that VAEs can effectively extract interpretable latent features from sparse high-throughput biological data [18]. Given the limited availability of experimental data, we employ a generative model with noise injection to generate synthetic samples that mimic real data. We then use a VAE to learn the underlying distribution of the augmented data, capturing its complexity and producing new test data points that further augmented training datasets. These augmented training datasets improve predictive performance and verify overall model robustness.

For dimensionality reduction, we assess several techniques, including t-distributed stochastic neighbor embedding (t-SNE), principal component analysis (PCA), and feature importance analysis using random forest algorithms, with the objective of enhancing model interpretability and computational efficiency. Dimensionality reduction is essential in microbiological studies since microbial community data typically have far more taxa than samples, making dimensionality reduction “a key component” for visualization and analysis of complex microbiome data [19]. As neural networks require numerical input, categorical environmental variables are transformed into numerical representations using autoencoders, which effectively encode geographic coordinates and categorical data into decodable numerical embeddings. This process enables dimensionality reduction while preserving essential information necessary for model training and interpretability. Consistent with this, Oh et al. used autoencoders to generate a low-dimensional representation of thousands of microbial markers, noting that such compression is required to “handle the high-dimensional data with low sample sizes” [20].

In the context of high-dimensional microbial composition data, conventional dimensionality reduction methods may result in the loss of biologically meaningful features embedded within these complex community profiles. To mitigate this, we prioritize feature importance analysis derived from random forest models, which offers a more biologically informed reduction strategy by preserving critical variables. Prior studies have demonstrated that random forest-based feature selection rankings provide robust, biologically meaningful variable importances for microbiome classification [21]. Subsequently, a neural network is trained on the augmented dataset to predict oil contamination levels from microbial profiles, and the model’s robustness is probed using VAE-generated synthetic stress test samples.

Our study introduces a compact and robust machine learning framework designed to address the challenges of high-dimensional, small-sample microbial datasets with several advantages. First, it encodes categorical environmental information (e.g., location, oil type) into numerical form that can be decoded back into interpretable textual labels. Second, to enhance model interpretability and efficiency, we apply random forest-based feature selection, which identifies biologically relevant microbial species correlated with oil contamination. This method outperforms conventional dimensional reduction techniques such as PCA and t-SNE by preserving critical biological variables. Third, to address data scarcity, we generate realistic synthetic microbial community datasets using a hybrid approach that combines a noise-injected data augmentation with a variational autoencoder (VAE). The VAE captures the latent structure of augmented data and generates diverse and representative samples used only for synthetic stress testing of model robustness. Fourth, our model achieves high predictive accuracy (R² up to 0.99) using a small subset (3–10) of microbial species, substantially simplifying analysis and reducing computational cost. The model shows strong internal consistency on VAE-generated synthetic stress-testing samples, but broader generalization to unseen environmental conditions requires evaluation on larger, independent datasets with formal validation procedures. Finally, our streamlined approach is model-independent, scalable, and applicable to both low- and high-dimensional datasets, offering a promising proof-of-concept solution beyond the constraints of traditional ecological modeling.

## Methods

### Training data and data processing

We collected microbial community data (Table 1) from multiple locations across three prior experiments, including Lake Michigan, Lake Superior, the Great Lakes Research Center at Michigan Technological University, and the Straits of Mackinac. The samples consist of oil-amended microcosms of lake surface water, from which community 16S rRNA genes were sequenced and taxonomy was assigned based on amplicon sequence variants (ASVs). For brevity, the details of the laboratory methods are provided in the Supplementary Information. One of the experiments is described in Byrne et al. 2021 [22]. Notably, the three experiments used slightly different methods for sequencing, collection times, and geographic locations. This provides a greater range of data variability than a single sampling effort would have, resulting in a more generalizable dataset.

**Table 1:** Raw datasets, including environmental and biological measurements, were collected at various locations and times. Variables comprise encoded location coordinates (latitude and longitude), temperature (°C), encoded oil types, and bacterial counts for identified species. Data was collected at monthly intervals. The “Location” and “Oil Type” columns are unitless encoded values generated using autoencoders. The “Month” column is encoded as (10 × month); for example, 80 corresponds to August. Columns labeled “Bac 1” to “Bac 503” represent the counts of bacteria species detected during sampling. The complete training dataset is provided in the supplementary information.

Location	Time (10 × weeks)	Oil Type	Temp (°C)	Month (10 × month)	Bac 1	Bac 2	…	Bac 503
10.39	30	45.11	23	80	116	58	…	0
10.39	50	146.27	23	80	57	35	…	0
…	…	…	…	…	…	…	…	…
42.83	60	146.27	23	50	0	0	…	0
42.83	70	146.27	23	50	1	0	…	0

The dataset includes the following features as inputs for the augmented data NN: location, incubation time, oil type, temperature, month, and 16S rRNA taxonomic composition (Table 1). The first column contains the encoded location value derived from the latitude and longitude of the samples. The second column denotes the incubation time, ranging from week 0 to week 7 (multiplied by 10 in data processing). Temperature represents the lab conditions during incubation. The month column indicates the season when the samples were collected (also multiplied by 10 in data processing). The columns labeled “Bac 1” to “Bac 503” correspond to the compositions of 503 different bacterium types in each sample. Thus, each row in the dataset represents an independent sample, encapsulating its geographic, temporal, and microbial characteristics.

### Autoencoder

**Fig 1:**
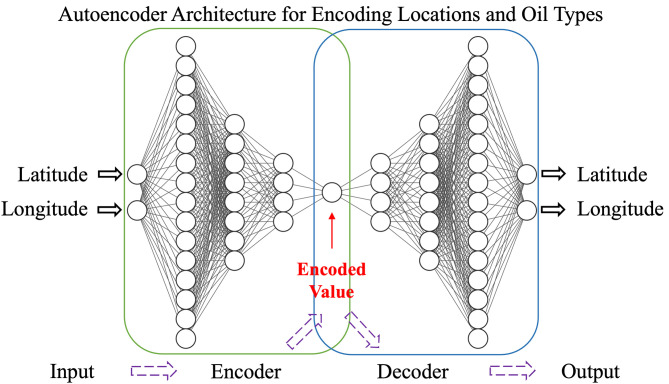
Schematic representation of an autoencoder for encoding “Location” (latitude and longitude) and “Oil type”. The encoder compresses the input into a reduced-dimensional, unitless encoded value, which the decoder then reconstructs into the original format.

An autoencoder is an unsupervised learning algorithm designed to replicate its input at the output layer (Fig 1). It consists of two parts: an encoder that compresses the input data into a lower-dimensional representation and a decoder that reconstructs the original data from the compressed form. In this study, the central hidden layer of the autoencoder contains a single neuron encoding latitude, longitude, and oil type into a compressed form. This setup reduces the dimensionality of the input features, simplifying data representation while preserving essential information.

Since the oil type information is categorical (“no oil,” “crude,” or “diesel”), it is first converted into binary digits: (0, 0) for “no oil,” (0, 1) for “crude,” and (1, 0) for “diesel.” These binary digits serve as inputs to the autoencoder, producing encoded oil-type values. Similarly, latitude and longitude are input into the autoencoder to generate encoded location values.

The location autoencoder is structured as follows: an input layer with 2 neurons, followed by hidden layers with 16, 8, and 4 neurons using ReLU activation. The bottleneck layer consists of a single neuron with sigmoid activation to effectively distribute the encoded latitude and longitude values, considering the narrow data range from Lake Michigan-based sampling. The decoder mirrors the encoder with hidden layers of 4, 8, and 16 neurons using ReLU activation, ending with an output layer of 2 neurons employing linear activation.

The oil-type autoencoder follows a similar architecture: an input layer with 2 neurons, hidden layers with 8 and 4 neurons using ReLU activation, and a bottleneck layer with a single neuron using ReLU activation for the encoded value. The decoder mirrors the encoder with hidden layers of 4 and 8 neurons using ReLU activation, ending with an output layer of 2 neurons using sigmoid activation, which is suitable for the binary (0 or 1) oil-type values. Both autoencoders utilize the Adam optimizer and mean squared error as the loss function, with a batch size of 16.

The encoded location and oil type retain the original latitude, longitude, and oil type information from the samples. After predicting oil contamination, the oil-type decoder determines whether the sample is oil contaminated and identifies the specific type of oil.

### Dimensionality reduction

**Fig 2:**
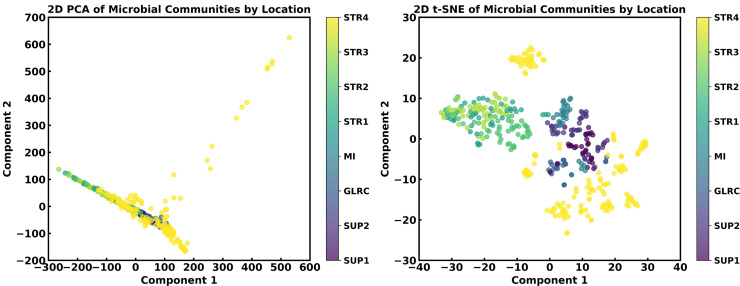
Visualization of microbial community distributions using PCA (left) and t-SNE (right). Each data point represents a microbial sample, colored by location. PCA captures variance linearly, while t-SNE reveals non-linear relationships. In the case of t-SNE, distinct dispersal patterns are observed across locations. STR, MI, GLRC, and SUP correspond to the Mackinaw Straits, Lake Michigan, the Great Lakes Research Center at Michigan Tech, and Lake Superior, respectively.

**PCA and t-SNE:** The bacterial compositions of each sample form a 503-dimensional dataset, representing measurements from 503 bacterial species. To maximize the use of all bacterial compositions while reducing noise in high-dimensional data and improving computational efficiency, we apply dimensionality reduction techniques. **Principal component analysis (PCA),** a linear reduction method, condenses the 503-dimensional data into lower-dimensional representations, such as two-dimensional PC1 and PC2, which capture key variations in bacterial composition. Additionally, t-distributed stochastic neighbor embedding (**t-SNE**), a nonlinear technique, is used to visualize complex patterns in the data. Both methods transform the high-dimensional feature space into two dimensions, facilitating visualization and pattern interpretation (Fig 2).

### Feature Importance with Random Forest Regressor (RFR)

Alternatively, we employ feature importance analysis using RFR to identify key features predictive of the oil type of samples. RFR is an ensemble learning method that constructs multiple uncorrelated decision trees during training and averages their outputs for regression tasks. Each decision tree of RFR iteratively splits the data by selecting the feature and threshold that best reduces impurity, quantified by the mean squared error (MSE). Feature importance is calculated based on the reduction in MSE at each split, where larger reductions indicate higher importance [23]. These importance values are aggregated across all trees to rank features according to their overall contribution to the model’s predictions. Interested readers are referred to the Supplementary Information for additional details on how random forest ranks bacterial genera by importance.

We implemented RFR models using the RandomForestRegressor function from the scikit-learn library [24], which offers a robust suite of machine-learning algorithms. Feature importance is extracted via the “feature_importances_” attribute. To optimize performance, we tune hyperparameters, setting n_estimators = 2500 (number of trees) and max_depth = 25 (to limit tree levels for preventing overfitting). The random forest hyperparameters were selected empirically to balance predictive accuracy and computational cost. Increasing the number of trees beyond 2500 or the maximum depth beyond 25 did not improve predictive performance, while smaller values led to reduced model stability. Feature importance analysis was then used to identify bacterial taxa that most strongly influence oil-type prediction. Although the model was initially trained on all 503 features, using only the top 10 ranked features still yielded a training R² of approximately 0.904, with minimal loss in performance. This indicates that these top-ranking bacterial genera contain the majority of biologically relevant information required for oil-type differentiation, allowing accurate prediction while substantially reducing model complexity.

When dimensionality is reduced to two features, RFR feature importance identifies the original microbial genera that drive the predictions and maintains performance comparable to PCA and t-SNE while preserving biological interpretability (Table 2). With additional top-ranked features, the RFR approach can outperform PCA and t-SNE, as discussed later. The performance outcomes of these analyses are summarized in Figure S1 of the S1 File.

**Table 2:** Performance outcomes of PCA and t-SNE, and random-forest feature selection (top-2 features).

Number of Bacteria Features	ADNN Training R^2^	VAE Training R^2^	Oil Prediction Model Training R^2^	Oil Prediction Test R^2^ (VAE-Generated Stress Test)
PCA components (PC1 and PC2)	0.89	0.954	0.995	0.853
t-SNE components (t-SNE1 and t-SNE2)	0.943	0.980	0.952	0.866
Random forest feature selection (top 2)	0.838	0.995	0.995	0.834

### Data augmentation - augmented data neural network (ADNN)

Obtaining experimental samples and determining their bacterial compositions is a time-consuming process. As a result, sample limitations often occur, significantly restricting the amount of training data available for machine learning models. This issue is frequently addressed using transfer learning methods, where pre-trained machine learning models transfer knowledge acquired from other tasks in a source domain to target tasks. However, if the source and target domains are too dissimilar, the model may fail to generalize effectively, resulting in poor performance on the target task. More broadly, generative models have emerged as a promising approach for augmenting sparse ecological datasets. For example, Rafiq et al. discuss the use of generative models to enhance data-limited ecological studies [25].

To address data limitations and develop a more adaptable method for diverse bacterial composition datasets in oil-contaminated aquatic environments, we implemented a data augmentation network. This network consists of a traditional feedforward artificial neural network and generates additional training data that closely resemble the original experimental data. The input features include five sample attributes: encoded locations, incubation time, oil type, temperature, and month. Temperature represents the incubation conditions in the lab, while the month indicates the time of sample collection, capturing seasonal variations. The network outputs bacterial compositions processed after dimensionality reduction.

All samples were used to train the ADNN. The architecture (Fig 4) consists of 5 input neurons, 5 hidden layers, and *N* output neurons, where *N* is the number of selected bacterial features. The hidden layers are structured as follows: the first and fifth hidden layers contain 32 neurons with ReLU activation functions, the second and fourth hidden layers have 64 neurons with ReLU activation functions, and the third hidden layer consists of 128 neurons with Gaussian activation functions. The output layer employs a linear activation function. The model is trained for 3000 epochs with a batch size of 32. During training, we track the loss function and the training R² value every 100 epochs, saving the model with the highest training R² value.

To train the augmented data NN to produce synthetic data that closely resembles real data, we use a method known as noise injection in machine learning [26,27]. We start with 404 collected samples, each containing five features: location, time, oil type, temperature, and month. Based on these inputs, the network learns to predict the top-selected bacterial compositions.

To incorporate controlled randomness into the inputs, we calculate the mean and standard deviation ($\sigma_{\text{1}}$, $\sigma_{\text{2}}$, $\sigma_{\text{3}}$, $\sigma_{\text{4}}$, $\sigma_{\text{5}}$) for each feature. We then generate a random matrix of the same dimensions (404 × 5), where each feature column follows a normal distribution with mean zero and a standard deviation of 0.01$\sigma_{i}$. Adding this random matrix to the original input data creates 404 synthetic samples. This process is repeated to generate a total of 1,616 synthetic samples. Combined with the original 404 real samples, the final training set consists of a total of 2020 samples, which will be used to train a variational autoencoder introduced in the next subsection.

Noise-injected data should not be used for validating datasets to ensure an unbiased and accurate evaluation of model performance on real-world, unseen data. Introducing noise can distort this assessment by altering the data distribution, introducing potential biases, and skewing the evaluation metrics, leading to unreliable and non-representative results. Since the augmented data NN aims to generate data similar to the real samples, achieving a higher training R² value is critical. Therefore, the entire dataset is used to ensure the generator learns all available information about microbial degradation systems.

### Latent-space perturbation analysis using a variational autoencoder (VAE)

In this study, VAE-generated samples are not intended to represent truly out-of-distribution ecological conditions. Instead, the VAE learns a smooth latent representation of the empirical data distribution (augmented for stability), from which controlled perturbations can be generated. These samples remain within the learned data manifold and are used to probe the robustness and internal consistency of the predictive model under biologically plausible variations.

VAEs can learn the probabilistic distribution of training data in their latent space by capturing mean values (μ) and standard deviations (σ) [28–30]. By sampling from the learned latent distributions, VAEs generate data similar to the original but not identical, ensuring variability and diversity in outputs. Compared to GANs, VAEs offer advantages such as requiring less training data and shorter training times and having a simpler architecture.

**Fig 3:**
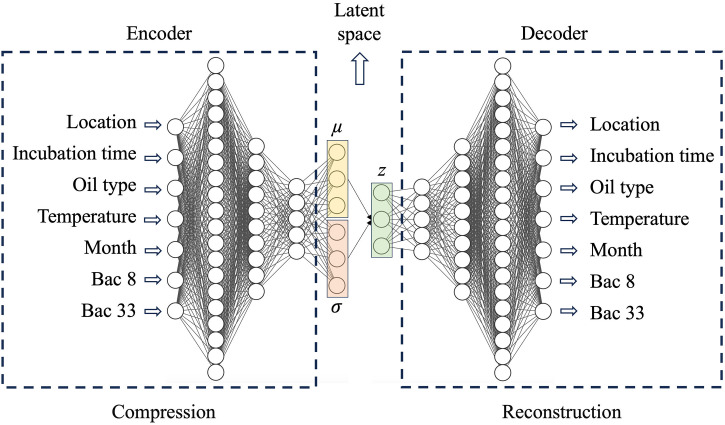
Architecture of variational autoencoder (VAE) used for data compression and reconstruction. The encoder maps input features (e.g., location, oil type, temperature, bacterial counts) into a latent space represented by a mean vector (μ) and standard deviation vector (σ), which characterizes the learned distribution. Using the reparameterization trick, a latent vector (z) is sampled and passed to the decoder to reconstruct the original input data or generate new realistic data points for synthetic stress testing.

The VAE architecture (Fig 3) consists of an encoder, a latent space, and a decoder. The encoder outputs μ and log-σ; a latent vector z is sampled using z=μ+σ·ε, where ε~N(0,I), and the decoder reconstructs the inputs. The loss combines reconstruction mean squared error and Kullback–Leibler divergence and is optimized with Adam for 3000 epochs with a batch size of 32. Inputs have (5 + *N*) neurons (location, incubation time, oil type, temperature, month, and the top *N* microbial features), followed by three dense layers of 256, 128, and 64 neurons with ReLU activation, a 3-dimensional latent space, and a sampling layer.

We monitor the R² value every 100 epochs during the training process and save the best model with the highest R² value. When well-trained using 2020 samples (404 real samples + 1616 synthetic samples), the VAE models the empirical training distribution of sample features and bacterial compositions. We then sample 2020 data points from the latent space to generate synthetic samples for stress testing the oil contamination prediction model. We use VAE-generated samples as controlled, in-distribution perturbations to probe internal robustness. True generalization to unseen real data is limited by sample size.

### Oil contamination prediction neural network

**Fig 4:**
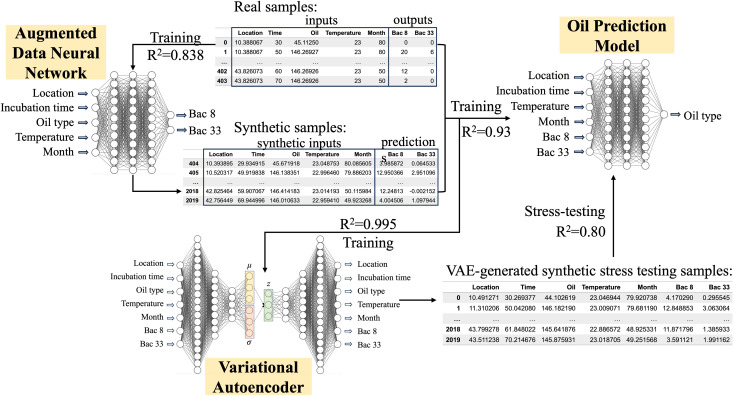
Machine learning framework for modeling microbial communities and predicting oil contamination. The RFR reduces the complexity of the 503-dimensional bacterial count data by identifying key bacterial features. The augmented data neural network is employed to create synthetic data to supplement real samples. The VAE learns the empirical feature distribution and produces additional synthetic samples for stress testing. The oil contamination prediction model achieves high accuracy when trained on both real and synthetic data, enhancing the robustness and reliability of predictions.

For predicting oil contamination, we use a neural network with (4 + *N*) input neurons (location, incubation time, temperature, month, plus *N* selected bacterial features), 5 hidden layers, and 1 output neuron for oil type prediction, as shown in Fig 4. The hidden layers are structured as follows: 32 neurons with ReLU activation functions, 64 neurons with ReLU activation functions, 128 neurons with ReLU activation functions, 64 neurons with ReLU activation functions, and 32 neurons with ReLU activation functions combined with batch normalization (BN). The model is trained using the Adam optimizer with mean squared error as the loss function for 1500 epochs and a batch size of 16.

BN is applied to mitigate overfitting, which is particularly beneficial when training with noisy data and a large dataset. We also investigated the effect of BN in our previous study [31]. During training, we save the best model with the highest R² value at each epoch. Since our augmented training set consists of 2020 samples (a combination of real data and synthetic data generated by the augmented data NN), BN helps improve the model’s performance by addressing noise and ensuring robust learning. By using BN, we achieve better performance and prevent overfitting, enhancing the neural network’s ability to accurately predict oil contamination based on the provided input features.

### Synthetic stress tests and data usage

To maintain separation between training and stress-testing datasets, the predictive neural network was trained using augmented data produced by the Augmented Data Neural Network (ADNN), while the stress-testing samples were independently generated from the latent space of the Variational Autoencoder (VAE). The VAE was trained on the augmented dataset to capture the underlying community distribution. In this design, VAE-generated samples serve as synthetic stress-testing data, which is biologically realistic but not directly reused from the training process.

Model performance is summarized by R² on real samples from the study cohort. Given the limited cohort, we prioritized model parsimony and training regularization. We used batch normalization and stochastic noise injection to mitigate overfitting during training. Performance summaries reflect internal fits and are presented as preliminary indicators rather than definitive out-of-sample accuracy. ADNN-generated synthetic samples were used to expand the training set, while VAE-generated samples served as synthetic stress tests for assessing internal consistency of the neural network pipeline, not as independent validation data.

### Limitations

The small sample size limits the reliability of performance estimates and prevents strong claims about generalizability. Generated samples cannot establish independent validation and may not capture the full range of biological variability. Findings should be viewed as a feasibility demonstration that requires confirmation on larger independent cohorts with formal validation procedures. Reported R² values are internal estimates. Because both ADNN and VAE are trained on the same limited cohort, evaluation on VAE-generated samples may lead to optimistic performance estimates; therefore, these results are interpreted strictly as internal robustness analyses rather than independent measures of generalization.

Sampling was restricted to the Great Lakes region during a limited seasonal window. Community composition and physicochemical drivers vary across watersheds; therefore, models trained here may capture region-specific signals. The associations reported should be viewed as hypotheses requiring external validation across independent regions and seasons.

ADNN augmentation (noise-injection method) was applied to the training set only to generate synthetic training samples. No augmentation was performed on the test set. The oil prediction NN was trained using the augmented training data. The same model architecture and hyperparameter configuration described above were applied without additional tuning for the hold-out experiment. Model performance was evaluated on the untouched held-out test set using R^2^.

## Results and discussion

### Autoencoder

We utilized autoencoders to convert oil type into numerical values and to encode latitude and longitude into single numerical values. The R² value for the oil type encoding and decoding process is 1, demonstrating that the oil type can be encoded and decoded with perfect precision. Similarly, the location can be decoded back to latitude and longitude. The training R² value for the location autoencoder is 0.989, indicating that latitude and longitude can be encoded and decoded with very high accuracy.

After the neural network models make predictions, a decoder translates the encoded oil type back into binary digits, which can then be translated into textual information. This information indicates whether the sample is oil-contaminated and, if so, whether it is contaminated by crude oil or refined oil. In this decoding process, (0,0) represents no oil in the sample, (0,1) represents contamination by crude oil, and (1,0) represents contamination by refined oil.

### Dimensionality reduction using random forest

As discussed in the previous section, PCA and t-SNE are not well-suited for this study due to limitations in interpretability. PCA performs a linear transformation to reduce dimensionality, producing principal components (e.g., PC1 and PC2) that represent linear combinations of the original variables. However, these principal components lack direct interpretability in a biological context, as they do not correspond to specific microbial compositions in the real world. Similarly, t-SNE, a stochastic and nonlinear dimensionality reduction method, presents two major limitations: (1) it is not reversible, meaning the original microbial information cannot be reconstructed, and (2) its output lacks biological interpretability, making it challenging to translate prediction results into meaningful microbiological insights.

Although alternative dimensionality reduction approaches occasionally yield higher test R^2^ values, RFR was preferred due to its biological interpretability, stability across feature counts, and direct mapping to microbial taxa. This enables mechanistic interpretation and hypothesis generation, which are central goals of environmental microbiome studies. The random forest model was trained using 503 bacterial composition features as input and the encoded oil type as output. The model achieved a training R² value of 0.904 across 404 samples (see code snippet in Supplemental Information).

**Fig 5:**
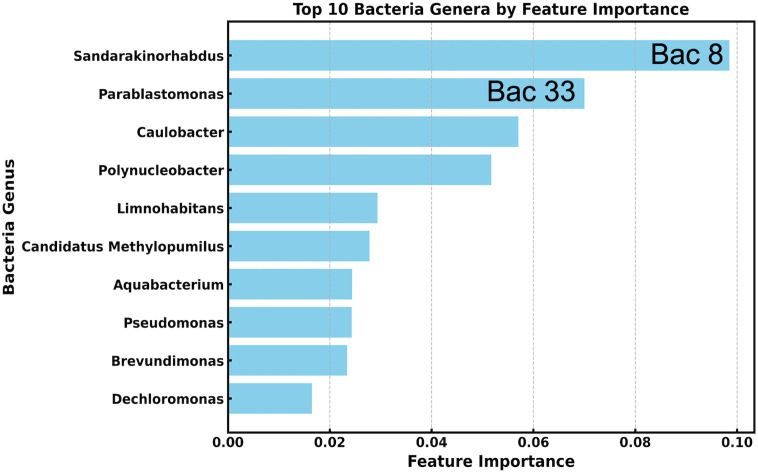
Horizontal bar plot of normalized feature importance scores from the RFR. The bars indicate the contribution of each genus to predicting oil types.

Fig 5 shows the ranked features that contribute most to predicting oil type. Unlike PCA or t-SNE, the random forest provides feature importance scores that directly identify the most significant bacterial genera for prediction. Using predictions from the trained RFR model, we found that selecting the top 3–10 features resulted in only a minimal reduction in prediction accuracy compared to using all 503 features. This suggests that the top-ranked features effectively capture the key characteristics of the full 503-dimensional bacterial composition data. The random forest approach offers interpretable results and identifies strong microbial indicators of oil contamination, providing meaningful biological insights. In this context, random forest outperformed PCA and t-SNE in dimensionality reduction. We therefore suggest feature importance as a dimensionality reduction method for microbial 16S rRNA taxonomic analysis.

### Prediction performance

**Fig 6:**
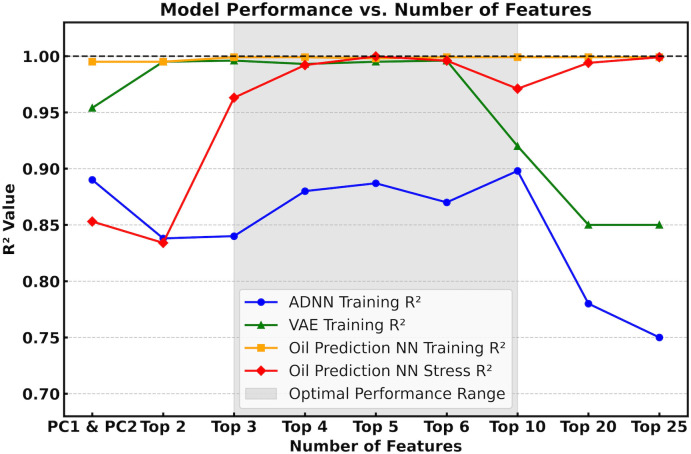
Comparison of R^2^ values across different feature sets. The augmented data NN (ADNN), VAE, and oil prediction model achieve optimal performance within the top 3 to top 10 feature range, exhibiting outstanding R² values exceeding 0.95 for both oil prediction and VAE.

Fig 6 presents the training results for different types of taxonomic compositions and compares the performance of various feature selection methods within a machine learning framework for oil prediction. Notably, using only the top two features yielded prediction accuracy comparable to that achieved with PCA-transformed data, demonstrating the efficiency of the feature selection approach. As the number of selected features increases, the training R² of the oil prediction model improves, reflecting the incorporation of more relevant information.

However, the training R² of the ADNN may decline when attempting to predict high-dimensional outputs from a limited set of input features, a task that poses significant challenges for neural networks. Importantly, the training R² of the ADNN also serves as an indicator of synthetic sample quality: higher R² values suggest that the synthetic data closely resemble real microbial community compositions, indicating higher fidelity. Improved sample quality enhances the VAE’s ability to learn meaningful distributions, thereby increasing the realism of VAE-generated samples and ultimately improving predictive performance in the oil prediction model.

There is a trade-off in selecting the number of community composition features. In this study, we observe that the training R² values of both the ADNN and the VAE remain stable initially as the number of top features increases, but begin to decline when more features are included. This suggests that the ADNN struggles to accurately predict microbial community compositions from the five known environmental factors as the dimensionality increases.

Note that the oil prediction model performs best when using the top 3–10 features, yielding R² values above 0.95 for both training and stress-testing datasets, suggesting strong predictive performance. Additionally, given a sample’s location, incubation time, temperature, month of collection, and five community composition features, oil contamination can be effectively estimated using both synthetic data from the ADNN and stress-testing data generated by the VAE. However, when the number of top features increases to 30, the ADNN shows a marked decline in predictive performance, indicating the limitations of modeling high-dimensional outputs with limited input features. Further details on model performance across different feature sets are provided in Table S1 of the S1 File.

Additionally, when our ADNN fails to effectively model real data (e.g., training R² < 0.75), the synthetic data it generates become less reliable. This issue is particularly evident when analyzing datasets with many microbial features, where complexity surpasses the learning capacity of the ADNN. As a result, the VAE trained on this less reliable augmented data produces unrealistic stress-testing samples, negatively impacting subsequent analyses.

**Fig 7:**
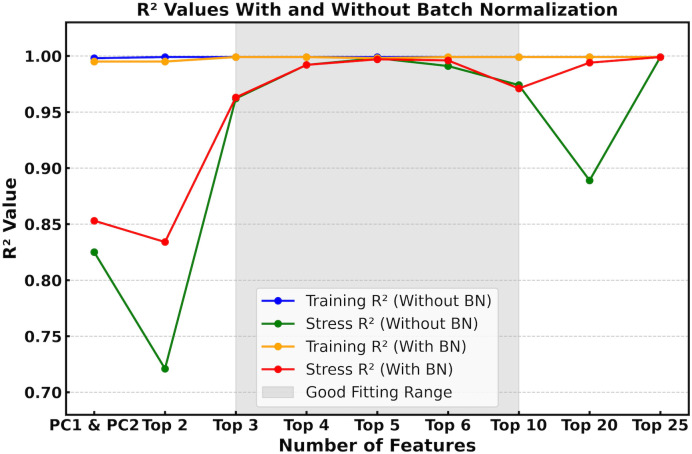
Comparison of training and stress-testing R² values for the oil-type prediction model with and without batch normalization (BN) across different feature sets.

In a well-generalized model, the training R² is typically equal to or slightly higher than the stress-testing R². A large discrepancy, with high training and low stress-testing R², indicates overfitting, while low values for both suggest underfitting. As shown in Fig 7, this training versus stress testing comparison indicates robust performance within the optimal range from top 3 to top 10 features, while overfitting is apparent outside this range. To address this issue, we applied batch normalization (BN) [31,32], a technique that reduces internal covariate shift by normalizing layer inputs across each mini-batch. This normalization improves gradient flow, enabling faster and more stable training.

Overall, BN enhances stress-testing performance by reducing overfitting and stabilizing learning, particularly for high-dimensional datasets involving 10 or more features. These findings underscore the combined importance of BN and effective feature selection in improving the accuracy and generalizability of oil contamination prediction models.

### Held-out evaluation and interpretation

To assess generalization to unseen real bottles, we performed a bottle-level hold-out evaluation. The dataset comprises 172 bottles (independent physical microcosms). We conducted 22 repeated random splits at an 80/20 bottle ratio, holding out approximately 34–35 bottles per split. The held-out test set contained about 83 real samples on average.

Under this protocol, the oil prediction neural network achieved high training performance in the hold-out experiment (training R^2^ = 0.9988), whereas performance on held-out bottles was lower and variable. Across the 22 splits, the mean test R^2^ was −0.150 (standard deviation 0.237; range −0.551 to 0.202). These results indicate that generalization to unseen bottles within this dataset is limited.

This pattern is consistent with the challenges of learning predictive relationships from a relatively small dataset (n = 404 samples distributed across 172 bottles) with substantial between-bottle variability. In this evaluation, ADNN augmentation via noise injection was applied to the training set only, and the held-out test set was not used in augmentation or model fitting. VAE-generated samples were not used for model evaluation; instead, the VAE component is used only for controlled perturbation analysis within the learned data manifold and should not be interpreted as independent validation or out-of-distribution testing.

## Conclusions

In this study, we developed a compact machine learning framework to predict oil contamination from limited experimental samples on microbial compositions in aqueous environments. Feature importance analysis using random forest efficiently reduced the complexity of the original 503-dimensional bacterial composition data, yielding more interpretable and tractable models. The high predictive accuracy achieved with only the top 3–10 community composition features suggests that these features capture the key taxa associated with responding to oil contamination. In particular, the random forest feature importance ranking (Fig 5) provides a ranked shortlist of candidate indicator genera that can guide mechanistic interpretation and targeted experimental validation in future studies.

The augmented data neural network (ADNN), combined with noise injection, generated synthetic data closely resembling real samples, thereby expanding the experimental dataset. This augmented data, along with the original samples, were used to train a neural network for oil-type prediction, achieving training accuracy of up to 99%. The optimal number of bacterial features was determined by evaluating the deviation between training and stress-testing accuracy, where the stress-testing data were generated from a variational autoencoder (VAE) trained on both synthetic and experimental data distributions. These VAE generated samples are used only for synthetic stress testing as controlled, in distribution perturbations and should not be interpreted as independent validation. Additionally, feature importance selection using random forest-based importance scores, combined with noise injection-based data augmentation, significantly reduced overfitting and improved predictive performance in our model.

Finally, we also note that batch normalization reduced overfitting and improved stress-testing performance, particularly in high-dimensional settings. Overall, this framework is applicable to both low- and high-dimensional data, offering a versatile approach for environmental monitoring and biodegradation applications. In the bottle-level hold-out evaluation, performance on held-out bottles (mean test R^2^ = −0.150 across 22 splits) was lower and variable than training performance, indicating limited generalization within this cohort. Although the current dataset is limited to samples from the Great Lakes region, such data constraints are common in environmental microbiology. Performance estimates reflect internal consistency within the sampled region and should be interpreted as region-specific, with generalizability to other regions remaining to be established. Future work should benchmark the full predictive pipeline against established classifiers on larger, multi-region datasets to quantify relative advantages.

## Supporting information

S1 File Supporting information. Contains supporting figures and tables. (ZIP)
