# Supplementary material for: Predicting oil contamination in water using machine learning on microbial compositions
Source: PLoS One. 2026 Mar 19;21(3):e0344571. doi: 10.1371/journal.pone.0344571 (PMC13001938; doi:10.1371/journal.pone.0344571)
Supplement: S1 File — (ZIP) [file pone.0344571.s001.zip › SI_Revised/SI_PLOS_final.pdf]

# Predicting Oil Contamination in Water Using Machine Learning on Microbial Compositions

## Supplementary Information

Tong Gao[1], Isaac Bigcraft[2], Stephen Techtmann[2,*], Issei Nakamura[1,*]

[1] Department of Physics, Michigan Technological University, Houghton, Michigan 49931, USA

[2] Department of Biological Sciences, Michigan Technological University, Houghton, Michigan 49931, USA

### 1. Experimental Method and Data Collection

The dataset consists of three separate experiments sampling oil-amended microcosms of water collected from seven locations across the Great Lakes. Surface water was collected at each site using a bucket and then stored in a 20 L carboy in an insulated ice-chest at approximately the ambient temperature of the water. Samples were returned to the lab within a week and stored at 4 °C until microcosm, which occurred within two weeks of sample collection. The three datasets from the three experiments have been internally termed 'MTU', 'Straits', and 'Seasons', with the Seasons dataset corresponding to Ref. [1]. The MTU and Straits dataset were collected in August 2016, and the Seasons dataset was collected in October 2018, May 2019, and July 2019. The sampling locations are shown in Figure S1.

In each experiment, microcosms were treated with oil to simulate exposure. The MTU and Straits experiments included three treatments: Control (no oil), Bakken crude oil, and Cold Lake Diluted Bitumen. The Seasons experiment used Control (no oil), Bakken crude oil, and non-highway diesel. For the purposes of model training, the heavy crude oil and non-highway diesel were considered as interchangeable. Oil-amended bottles contained a final concentration of 2.5 µl of oil in 100 ml of lake surface water to achieve 25 ppm.  Microcosms were incubated at room temperature in the dark. Samples from the October 2018 timepoint were incubated at room temperature, and a separate set of bottles was incubated at 4°C to test the impact of temperature on microbial response to oil exposure.

Sacrificial sampling of microcosm bottles occurred once per week for five weeks. At each time point, all of the water from each of the triplicate bottles for each condition (Bakken, Dilbit, and Control) was filtered through a PES filter (0.2 µm pore size, 47 mm diameter).  Filters were immediately frozen at -80 °C until sample processing.

Filters were cut in half using flame-sterilized scissors. One half of the filter was used for DNA extractions, while the other half was archived at -80 °C. DNA extractions were performed using either the Modified Miller Method [2] for the MTU and Straits experiments or the Zymobiomics DNA extraction kit for the Seasons dataset.

The 16S rRNA libraries for the MTU and Seasons experiments were prepared for sequencing of the V4-V5 region using the 515YR and 926R primer pair [3]. Sequencing adapters and indices were added using a second 8 cycle PCR following the Illumina 16S rRNA library preparation kit

protocol. Libraries were purified with the AxyPrep magnetic PCR clean up kit (Axygen). The purified amplicons were quantified using PicoGreen and pooled to a concentration of 4 nM. The Straits dataset was generated by sequencing the V4 region of the 16S rRNA gene using primers 515F and 806R as described in Ref. [4]. Sequencing of the libraries for all experiments was performed on an Illumina MiSeq.

To produce an amplicon sequence variant (ASV) table, we used the DADA2 pipeline found at https://benjjneb.github.io/dada2/tutorial.html. This pipeline involved filtering and trimming low-quality reads and learning the error rates for all three datasets to obtain denoised sequence variants. Based on the ASV table, chimeras were removed, and taxonomy was assigned to reads using the Silva v138.1 NR99 SSU database, providing the final taxonomic information used in our model.

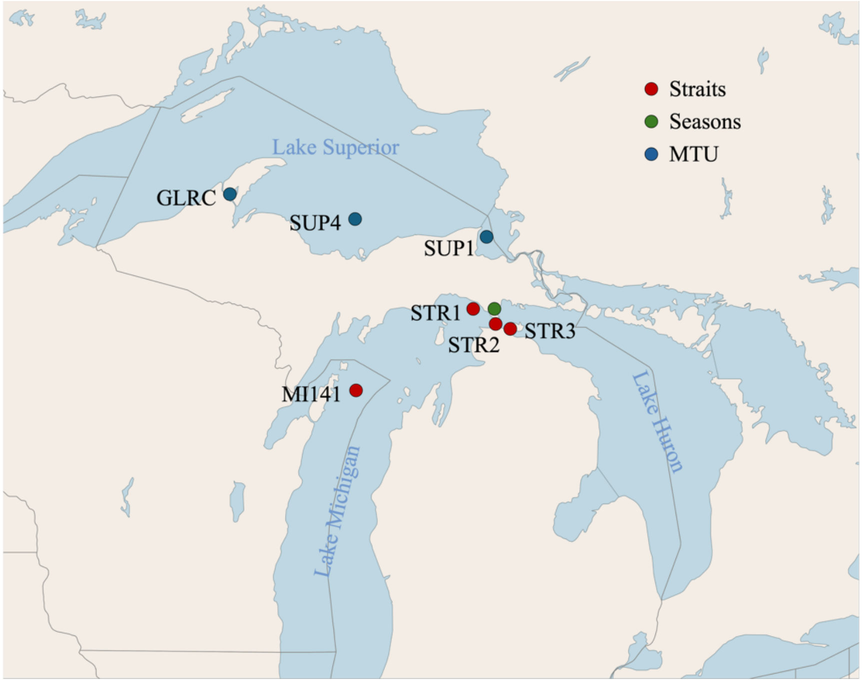


Figure S1 Map of sampling locations in the Great Lakes used for microbial community data. Labels indicate which of the three experiments sampled the location. Samples were collected from Lake Superior (SUP1, SUP4), Lake Michigan (MI141), the Great Lakes Research Center at Michigan Tech (GLRC), and the Straits of Mackinac (STR1, STR2, STR3). Basemap created using Natural Earth (public domain).

## 2. Random Forest Regressor

In this supplementary information, we explained how random forest regressor's feature importance ranks the most relevant bacterial genera based on their contributions to reducing mean squared errors. The training dataset containing microbial composition and environmental factors (503 features across 404 samples) is provided in SI_Dataset.csv.

Unlike classification trees, which use Gini impurity to determine splits, random forest regression (RFR) for regression problems uses mean squared error (MSE) as the split criterion. The MSE at a given node is calculated as:

$$MSE = \frac{1}{N}\sum_{i}(y_i - \bar{y})^2$$

where $y_i$ is the actual target value of the $i$-th sample at the node, $\underline{y}$ is the mean of all target values at that node, and $N$ is the number of samples at the node. To evaluate potential splits, the dataset is split into two child nodes, and their corresponding MSE values are computed. This criterion quantifies the variance of the target values within the node, and the algorithm selects splits that most effectively reduce this variance, thereby improving predictive accuracy.

The total MSE after a split is calculated as:

$$MSE_{split} = \frac{N_{left}}{N_{total}} MSE_{left} + \frac{N_{right}}{N_{total}} MSE_{right}$$

where $N_{left}$, $N_{right}$ are the number of samples in the left and right child nodes, respectively, and $MSE_{left}$ and $MSE_{right}$ are the corresponding MSEs.

The reduction in MSE from the split is given by:

$$\Delta MSE = MSE_{parent} - MSE_{split}$$

A positive and large ΔMSE indicates a beneficial split. The algorithm selects the feature and threshold at each split that yields the largest reduction in MSE and applies this process recursively to grow the tree.

Since RFR uses bootstrap sampling by default, each tree is trained on a randomly selected subset of the data. As a result, the root node of each tree contains fewer samples than the full dataset. For example, in one decision tree shown in Figure S2, Bac 3 was used for a split that reduced the MSE by 1179.2 ($\Delta MSE = 1179.2$), giving Bac 3 an importance score of 1179.2. In the next split, Bac 2 received an importance score of 774.9. This process is repeated for every feature at every split across all trees in the forest. The total importance score for each feature is then accumulated across all trees and normalized so that the scores sum to 1.0.

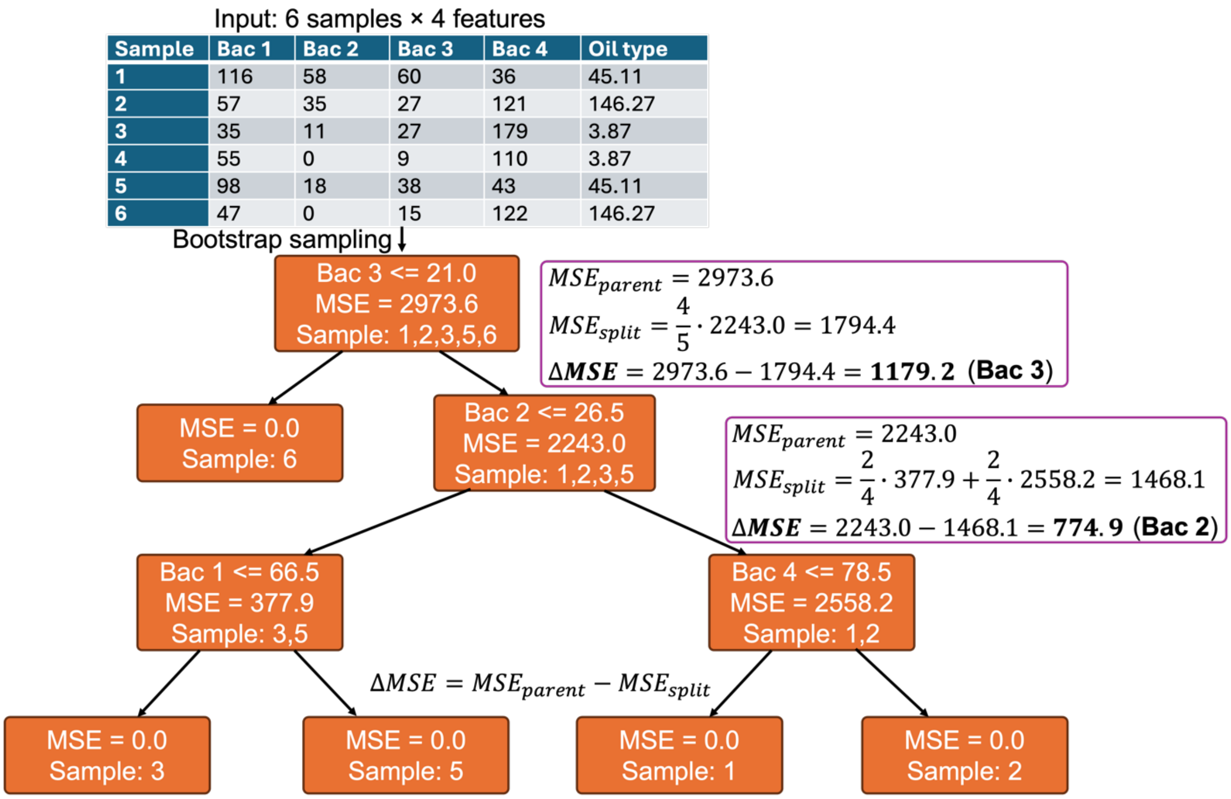


Figure S2 Diagram of feature importance calculation in a single decision tree. Mean squared error (MSE) reduction is computed at each split to assess feature importance. Each node shows the splitting condition, MSE, and sample names. Feature importance is determined by summing MSE reduction ($\Delta MSE$) across all splits and normalizing them.

We used an RFR implemented in the scikit-learn library to model oil contamination levels based on the full set of 503 bacterial composition features across 404 samples. The input matrix X included the 503 microbial composition features, while the output Y represented the encoded oil-type values for the 404 samples. The training $R^2$ score was 0.904, as shown in the code snippet below:

```
from sklearn.ensemble import RandomForestRegressor

from sklearn.metrics import r2_score
```

```
# Setup the Random Forest Regressor
rf_regressor = RandomForestRegressor(n_estimators=2500, max_depth=25,
random_state=48)

# Train model
rf_regressor.fit(X, Y)

# Make predictions on the training set
Y_pred = rf_regressor.predict(X)

# Calculate R² score
r2 = r2_score(Y, Y_pred)
print(f"R^2 Score: {r2}")
# Output: R^2 Score: 0.9037895021679457
```

Table S1 Performance of dimensionality reduction techniques and neural network models evaluated using the coefficient of determination ($R^2$). The table presents training $R^2$ values for the augmented data neural network (ADNN), VAE, and oil prediction neural network across different feature sets. Test $R^2$ values are shown for the oil prediction NN trained on VAE-generated samples. Optimal performance is observed with the top 3 to top 10 features. “NA” and negative values indicate poor performance due to insufficient training of the generator and VAE. Models with top 30 to full feature sets (top 503) exhibit significantly lower performance.

| Number of Bacteria Features | ADNN Training $R^2$ | VAE Training $R^2$ | Oil Prediction NN Training $R^2$ | Oil Prediction Test $R^2$ (VAE-Generated Data) |
|---|---|---|---|---|
| PCA components (PC1 and PC2) | 0.89 | 0.954 | **0.995** | **0.853** |
| Top 2 | 0.838 | 0.995 | **0.995** | **0.834** |
| **Top 3** | 0.840 | 0.996 | **0.999** | **0.963** |
| **Top 4** | 0.880 | 0.993 | **0.999** | **0.992** |
| **Top 5** | 0.887 | 0.995 | **0.998** | **0.997** |
| **Top 6** | 0.870 | 0.996 | **0.999** | **0.996** |
| **Top 10** | 0.898 | 0.920 | **0.999** | **0.971** |
| Top 20 | 0.780 | 0.850 | **0.999** | **0.994** |
| Top 25 | 0.750 | 0.850 | **0.999** | **0.999** |
| Top 30 | 0.740 | 0.000 | NA | NA |
| Top 40 | 0.700 | 0.000 | NA | NA |
| Top 50 | 0.650 | 0.000 | NA | NA |
| 503 | 0.100 | -19.000 | NA | NA |

## References

1. Byrne ER, Roche KM, Schaerer LG, Techtmann SM. Temporal variation of crude and refined oil biodegradation rates and microbial community composition in freshwater systems. Journal of Great Lakes Research. 2021;47(5):1376-85. doi: https://doi.org/10.1016/j.jglr.2021.08.003.
2. Techtmann SM, Fortney JL, Ayers KA, Joyner DC, Linley TD, Pfiffner SM, et al. The Unique Chemistry of Eastern Mediterranean Water Masses Selects for Distinct Microbial Communities by Depth. PLOS ONE. 2015;10(3):e0120605. doi: 10.1371/journal.pone.0120605.

3. Parada AE, Needham DM, Fuhrman JA. Every base matters: assessing small subunit rRNA primers for marine microbiomes with mock communities, time series and global field samples. Environmental Microbiology. 2016;18(5):1403-14. doi: https://doi.org/10.1111/1462-2920.13023.
4. Kapoor V, Li X, Elk M, Chandran K, Impellitteri CA, Domingo JWS. Impact of Heavy Metals on Transcriptional and Physiological Activity of Nitrifying Bacteria. Environ Sci Technol. 2015;49(22):13454-62. doi: 10.1021/acs.est.5b02748. PubMed PMID: WOS:000365151200042.
